# Long-Term Results of Kyocera Modular Limb Salvage System after Resection of Tumors in the Distal Part of the Femur: Report from Japanese Musculoskeletal Oncology Group Study

**DOI:** 10.3390/cancers14040870

**Published:** 2022-02-10

**Authors:** Tomoki Nakamura, Akihiko Matsumine, Yu Toda, Satoshi Takenaka, Hidetatsu Outani, Tomohiro Fujiwara, Yoshihiro Nishida, Satoshi Tsukushi, Yasunori Tome, Teruya Kawamoto, Munehisa Kito, Naohiro Shinohara, Masato Tomita, Tomoaki Torigoe, Akihiro Sudo, Hirotaka Kawano

**Affiliations:** 1 Department of Orthopaedic Surgery, Mie University Graduate School of Medicine, Tsu 514-8507, Japan; a-sudou@clin.medic.mie-u.ac.jp; 2 Department of Orthopaedics and Rehabilitation Medicine, Faculty of Medical Sciences, University of Fukui, Eiheiji 910-1193, Japan; matsumin@u-fukui.ac.jp; 3 Department of Musculoskeletal Oncology, National Cancer Center Hospital, Tokyo 104-0045, Japan; yutoda2@ncc.go.jp; 4 Department of Orthopaedic Surgery, Osaka International Cancer Institute, Osaka 541-8567, Japan; s.takenaka.0816@gmail.com; 5 Department of Orthopaedic Surgery, Osaka University Graduate School of Medicine, Suita 565-0871, Japan; h-otani@ort.med.osaka-u.ac.jp; 6 Department of Orthopaedic Surgery, Okayama University Graduate School of Medicine, Dentistry, Pharmaceutical Science, Okayama 700-8558, Japan; tomomedvn@gmail.com; 7 Department of Rehabilitation, Nagoya University Hospital, Nagoya 466-8550, Japan; ynishida@med.nagoya-u.ac.jp; 8 Department of Orthopaedic Surgery, Aichi Cancer Center Hospital, Nagoya 464-0021, Japan; s-tsuku@aichi-cc.jp; 9 Department of Orthopedic Surgery, Graduate School of Medicine, University of the Ryukyus, Nishihara 903-0215, Japan; yastome@med.u-ryukyu.ac.jp; 10 Department of Orthopaedic Surgery, Kobe University Graduate School of Medicine, Kobe 650-0017, Japan; trykwmt@med.kobe-u.ac.jp; 11 Department of Orthopaedic Surgery, Shinshu University School of Medicine, Matsumoto 390-8621, Japan; mune0527@yahoo.co.jp; 12 Department of Orthopaedic Surgery, Kagoshima University, Kagoshima 890-8520, Japan; sou.tadao@icloud.com; 13 Department of Orthopaedic Surgery, Nagasaki University Graduate School of Biomedical Sciences, Nagasaki 852-8520, Japan; mtomita@nagasaki-u.ac.jp; 14 Department of Orthopaedic Oncology and Surgery, Saitama Medical University International Medical Center, Hidaka 350-1298, Japan; ttorigoe@saitama-med.ac.jp; 15 Department of Orthopaedic Surgery, Teikyo University School of Medicine, Tokyo 173-8608, Japan; hkawano-tky@umin.net

**Keywords:** prosthesis, musculoskeletal tumors, distal femur, implant survival

## Abstract

**Simple Summary:**

We aimed to elucidate the long-term outcomes of a distal femur reconstruction system in 125 patients with bone and soft tissue tumors. Implant survival rates at 10 and 15 years were 58.5% and 39.4%. Stem breakage should be considered in patients with cementless and/or smaller femoral stem sizes. Aseptic loosening should be considered in patients with a cement system after 10 years.

**Abstract:**

Background: The distal femur is a common site of bone tumors. After surgical resection, prosthetic replacement is a major reconstruction method. We aimed to elucidate the long-term outcomes of the Kyocera Modular Limb Salvage (KMLS) systems after resection of tumors in the distal part of the femur. Methods: Between 1998 and 2014, 125 patients were treated at 14 institutions. There were 59 males and 66 females, with a mean age of 35 years. The mean follow-up period was 132 months. Results: There had been 65 additional surgeries, including 56 revisions and 9 amputations: 15 for aseptic loosening, 14 for stem breakage, 13 for deep infection, 13 for rotator-hinge bushing failure, 5 for local recurrence, and 5 for others. Implant survival rates at 10 and 15 years were 58.5% and 39.4%. The cumulative incidence of 15-year revision for femoral stem breakage was 31.7% in patients with cementless fixation. The 15-year cumulative incidence of revision for aseptic loosening was 19.8% in patients with cement fixation. Conclusions: KMLS systems represent a reliable system with long-term results. Stem breakage should be considered in patients with cementless and/or smaller femoral stem sizes. Aseptic loosening should be considered in patients with cement systems after 10 years.

## 1. Introduction

Limb salvage surgery has become more common for treating bone and soft tissue sarcoma in the extremities due to advances in surgical techniques, chemotherapy, and imaging modalities [1,2,3]. The distal femur is a common site of primary and metastatic bone tumors [4]. After surgical resection, prosthetic replacement is a major reconstruction method due to early weight bearing, immediate stability, and availability [5,6]. However, most types of prostheses are designed for the Caucasian body type and are frequently too large and heavy for Asian-Pacific patients. Therefore, the Japanese Musculoskeletal Oncology Group (JMOG) has developed an original prosthesis (KYOCERA Physio Hinge Knee system Type III (PHK III)) that requires bone cement to fix the femoral stem [5]. In 2002, the JMOG developed a new cementless stem in addition to the PHK III series and introduced the prosthesis as the Kyocera Modular Limb Salvage (KMLS) system [6]. In the KMLS system, we can choose between cemented and cementless stems according to the situation of the patients. Although short-to medium-term outcomes have been reported and satisfactory, further long-term analysis is required. Therefore, we aimed to elucidate the long-term outcomes of the KMLS systems after resection of tumors in the distal part of the femur. We define the KMLS systems to include the PHK III series because the design of PHK III series is the same as the cement-type KMLS systems.

## 2. Materials and Methods

### 2.1. Patients

This study was approved by the Clinical Research Ethics Review Committee of Mie University Hospital (H2020-174). Between 1998 and 2014, 125 patients were treated by surgeons of the JMOG using the KMLS systems at 14 institutions (Table 1). Records of all patients were collected using a questionnaire administered to members of the JMOG. There were 59 males and 66 females, with a mean age of 35 years (range, 9–79). The mean body mass index (BMI) was 21.3 kg/m^2^ (28 missing data). Primary malignant bone tumors included 85 conventional osteosarcomas, 9 undifferentiated pleomorphic sarcomas of the bone, 6 parosteal osteosarcomas, 5 chondrosarcomas, and 4 others. We also included seven giant cell tumors, seven metastatic bone tumors, and two soft tissue sarcomas. Chemotherapy was administered to 84 patients. Of the 125 patients, 119 underwent primary surgery. The remaining six patients underwent conversion surgery after other implant systems or reconstruction using autologous bone grafts. We excluded patients without any revisions within 5 years after surgery. The mean follow-up period after surgery was 132 months (median, 136 months). This study was approved by the institutional review board. Informed consent was waived due to the nature of this study.

### 2.2. Procedure for Tumor Resection

Of the 118 patients who underwent primary surgery, wide tumor resection was performed in 110 patients, and marginal tumor resection was performed in eight patients. Extra-articular resection was performed in 21 patients, while intra-articular resection was performed in 94 patients (unknown in three patients). More than three segments of the quadriceps femoris muscle were resected in 13 patients.

### 2.3. Prosthesis

**Figure 1:**
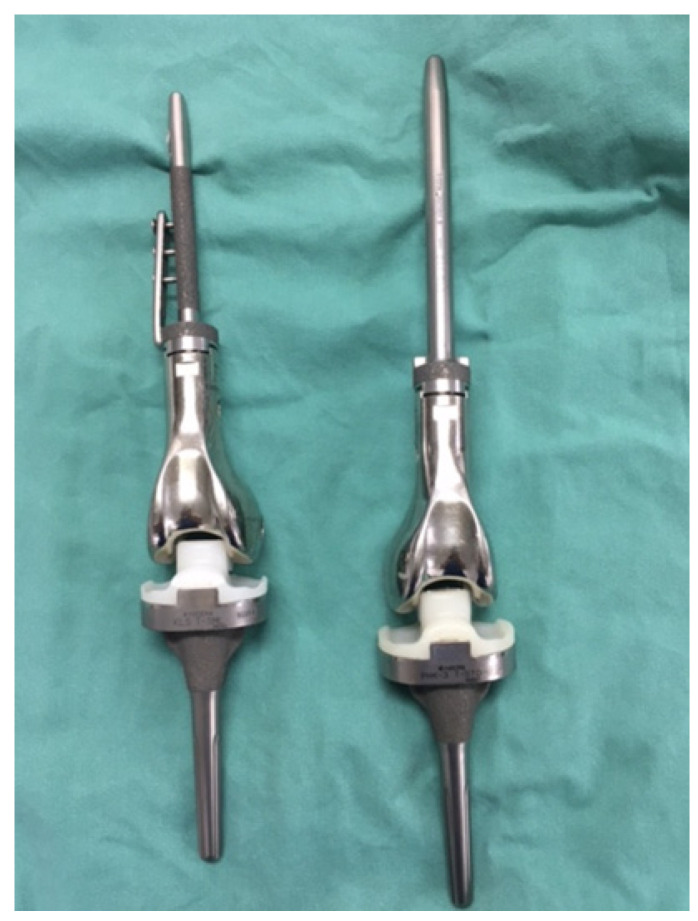
The detailed features of KMLS systems (Left, cementless system; Right, cement system). The cementless femoral stem has three unique non-penetrating holes and a side plate. The tibial component is the same in the cement and cementless types. The PHK III system is the same design as the KMLS cement system.

The KMLS system is a full modular prosthetic system with a rotating hinge joint and is designed for Asian patients with a smaller anatomical architecture of the knee joint (Figure 1). This system has a unique semirotating hinge joint that allows a maximal flexion of 142° and an internal/external rotation of 5°. The metallic parts of this system are made of lightweight and high-strength titanium alloy (Ti-6Al-4V) with good biocompatibility and biostability. The metallic surface of the hinge shaft and the rotator, which creates friction between high-density polyethylene, is fabricated using a surface-hardening treatment by azote-ionic impregnation to increase the durability of the hinge joint. The rotation sleeve, plate, and shaft sleeve were made of ultra-high molecular weight polyethylene. Cement-type systems require the use of polymethylmethacrylate cement for the fixation of the components of the femoral stem and tibia. In 2002, the cementless-type femoral stem was added to the series and has been chosen according to the situation of the patients. The cementless-type femoral stem component has three unique non-penetrating holes with a screw thread and a side plate with three screw holes (Figure 1). To achieve initial implant fixation, three side bolts can be inserted through the screw holes on the side plate. The interface of the femoral stem component is processed by porous proofing to promote bone ingrowth. The diameter of the femoral stem ranged from 10 to 13 mm in the cement type and from 12 to 15 mm in the cementless type. The custom-made 10 and 11 mm diameter femoral cementless stems were used in two patients. 

### 2.4. Reconstruction

Fifty-two patients underwent cement stem treatment, and seventy-three patients were treated with the cementless stem. The length of the femoral component ranged from 90 to 290 mm. The frequent lengths of the femoral component were 110 mm (*n* = 19), 130 mm (*n* = 23), 150 mm (*n* = 30), and 170 mm (*n* = 19). The femoral stem size ranged from 10 to 15 mm (Table 1). The patellar component was replaced in 21 patients. A local musculocutaneous flap was required in 10 patients, and a free vascularized flap was required in one patient.

### 2.5. Statistical Analysis

The relationship between patient characteristics and tumor characteristics was analyzed using the Mann–Whitney U-test for quantitative data and the chi-square test for qualitative data. The implant survival rate was estimated as the time from surgery to revision surgery due to implant failure. Implant failure was defined as replacement and/or removal of any part of the prosthesis due to local recurrence, polyethylene bushing failure, fracture, stem breakage, aseptic loosening, and infection. We also calculated implant survival, which defined the endpoint as metal component removal (major revision: removal of the femoral stem and/or tibial components). Survival curves were constructed using the Kaplan–Meier method. The log-rank test was used to compare the implant survival of patients. Statistical significance was set at *p* < 0.05. All statistical analyses were performed with the EZR graphical user interface (Saitama Medical Center, Jichi Medical University, Saitama, Japan) for R (R Foundation for Statistical Computing, Vienna, Austria), which is a modified version of R Commander designed to add statistical functions frequently used in biostatistics.

## 3. Results

At the time of the last follow-up, the patient’s status was as followed; continuous disease free (*n* = 96), no evidence of disease (*n* = 13), alive with disease (*n* = 5), died of disease (*n* = 10), and died of other disease (*n* = 1). There had been 65 additional surgeries (52% of the patients), including 56 revisions and 9 amputations (Table 2): 15 for aseptic loosening (9.1–190 months), 14 for stem breakage (6.6–159 months), 13 for deep infection (1.4–145 months), 13 for rotator-hinge bushing failure (e.g., wear of the rotation sleeve, breakage of tibial tray) (18.6–165 months), 5 for local recurrence (7.3–21.4 months), 2 for fracture (4.2 and 194 months) and 3 for others (5.3–108 months). Infection was related to the number of resections of the quadriceps femoral muscles (4/12 in 3 or 4 muscle resections versus 7/96 in 0–2 muscle resections) (*p* = 0.02).

**Figure 2:**
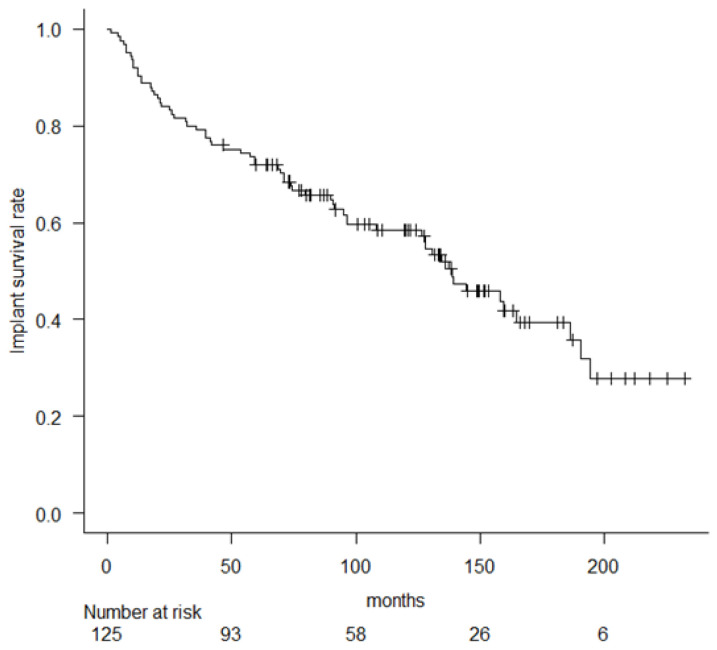
Kaplan–Meier curve showing implant survival rate. All causes of implant failure are included.

Implant survival rates at 5, 10, and 15 years were 72% (95% confidence interval (CI), 63.2–79), 58.5% (95% CI, 48.9–66.9), and 39.4% (95% CI, 28.3–50.3) (Figure 2). 

There was no significant difference in implant survival between the cementless and cement stem fixation groups. Implant survival rates at 5-, 10-, and 15-year in 73 patients with cementless fixation were 76.7% (95% CI, 65.2–84.8), 55.7% (95% CI, 42.9–66.8), and 35.3% (95% CI, 21.7–49.3), whereas 65.2% (95% CI, 50.6–76.5), 63.1% (95% CI, 48.3–74.6), and 46.4% (95% CI, 28.8–62.4) in 52 patients with cement fixation, respectively (*p* = 0.86). The log-rank test did not show statistical differences between the implant survival rate and the following variables: patient age, sex, BMI, resection of the joint capsule, administration of chemotherapy, patellar replacement, and number of resections of quadriceps femoral muscle (Table 3).

**Figure 3:**
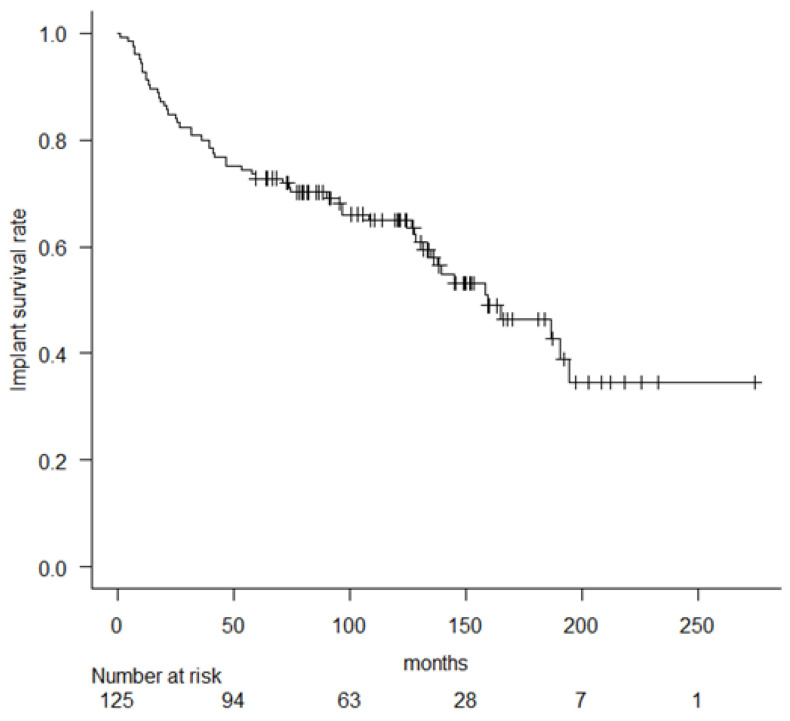
Kaplan–Meier curve showing implant survival rate (major revision).

Next, we estimated implant survival, which defined the endpoint as metal component removal (major revision), including the femoral component, stem and tibial components. Major revisions were performed, including 45 revisions and 9 amputations. The 5-, 10-, and 15-year implant survival rate (major revision) was 72.8% (95% CI, 64.1–79.7), 64.9% (95% CI, 55.5–72.8) and 46.3% (95% CI, 34.5–57.3) (Figure 3). 

**Figure 4:**
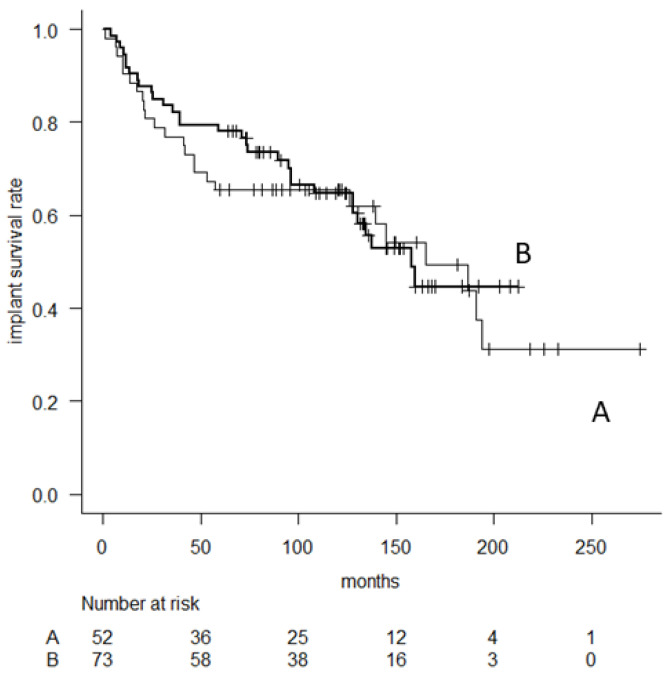
Kaplan–Meier curve comparing patients with cement (**A**) and cementless (**B**) femoral stem fixation.

No significant differences in implant survival were observed between the cementless and cement stem fixation groups. The 5-, 10-, and 15-year implant survival (major revision) in 73 patients with cementless fixation was 78.1% (95% CI, 66.7–86), 64.8% (95% CI, 52–75) and 44.7% (95% CI, 29.1–59.1), whereas 65.4% (95% CI, 50.8–76.6), 65.4% (95% CI, 50.8–76.6) and 49.3% (95% CI, 31.5–64.8) in 52 patients with cement fixation, respectively, (*p* = 0.71) (Figure 4).

**Figure 5:**
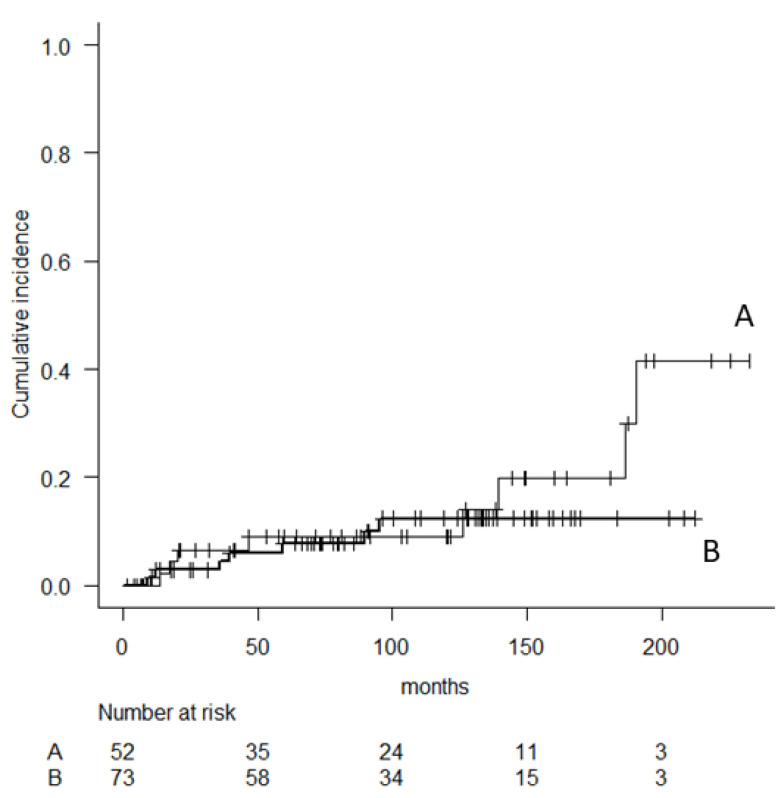
Cumulative risk analysis of implant failure due to aseptic loosening comparing patients with cement (**A**) and cementless (**B**) femoral stem.

**Figure 6:**
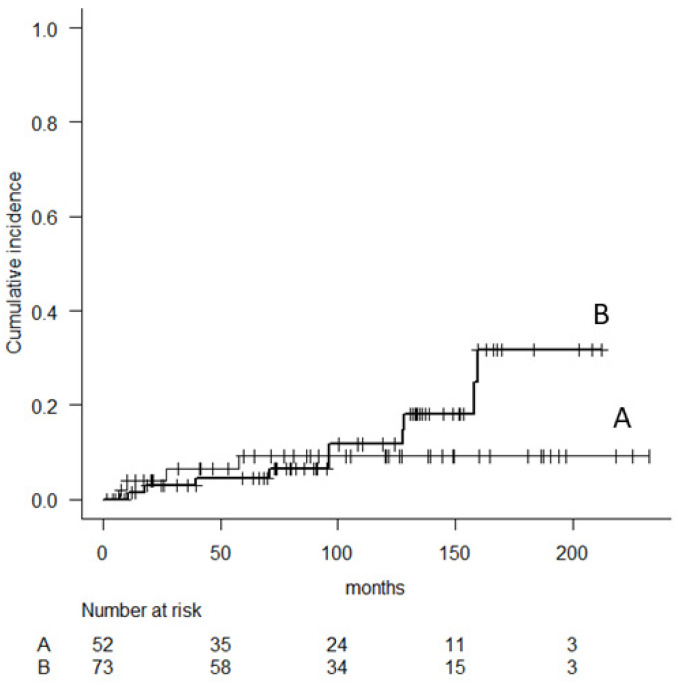
Cumulative risk analysis of implant failure due to stem breakage comparing patients with cement (**A**) and cementless (**B**) femoral stem.

There was no significant variable to predict major revision, although the administration of perioperative chemotherapy was a marginally significant variable (*p* = 0.06). The 5-, 10-, and 15-year implant survival (major revision) in 83 patients with perioperative chemotherapy was 67.5% (95% CI, 56.3–76.4), 60.4% (95% CI, 48.8–70.2), and 38.6% (95% CI, 24.4–52.6), respectively, whereas 83.3% (95% CI, 68.2–91.7), 74% (95% CI, 56.7–85.2) and 61.9% (95% CI, 42.4–76.4) in 42 patients without perioperative chemotherapy, respectively. The cumulative incidence of revision for aseptic loosening and femoral stem breakage was estimated (Figure 5 and Figure 6). Aseptic loosening was observed in six patients (8.2%) with cementless fixation and nine patients (17.3%) with cement fixation. The 5-, 10- and 15-year cumulative incidence of revision for aseptic loosening was 6% (95% CI, 2.3–15.3), 12.4% (95% CI, 6.0–24.7), 12.4% (95% CI, 6.0–24.7) in patients with cementless fixation and 9.1% (95% CI, 3.5–22.4), 9.1% (95% CI, 3.5–22.4), and 19.8% (95% CI, 13.1–59.2) in patients with cement fixation, respectively (*p* = 0.45). Analyses were performed to determine independent predictors of aseptic loosening, including age, BMI, sex, diameter of stem length, ratio of total length of prostheses (TLP)/stem length (SL), administration of chemotherapy, number of resections of the femoral quadriceps muscle, and use of cementation for femoral stem fixation. However, no significant variables were observed. 

Stem breakage was observed in 10 patients (13.7%) with cementless fixation and four (7.7%) with cement fixation. The cumulative incidence of 5-, 10- and 15-years revision for femoral stem breakage was 4.6% (95% CI, 1.5–13.5), 11.7% (95% CI, 5.3–24.8), 31.7% (95% CI, 16.2–55.9) in patients with cementless fixation and 9.1% (95% CI, 3.5–22.8), 9.1% (95% CI, 3.5–22.8), 9.1% (95% CI, 3.5–22.8) in patients with cement fixation, respectively (*p* = 0.36). Femoral stem breakage was observed between diameters of 10 and 13 mm. When we divided patients into two groups according to cement or cementless fixation, stem breakage was observed in patients with relatively thin stems: 10 mm (3 cases) and 11 mm (one case) in cement fixation, and 12 mm (8 cases) and 13 mm (2 cases) in cementless fixation.

Of the 56 patients who underwent revision surgery, reimplant failure was observed in 14 patients. Of the 14 patients, 10 underwent re-revision surgery due to the same cause of initial revision. Loosening recurred in seven patients.

Limb salvage rates at 5, 10, and 15 years were 95.2% (95% CI, 89.6–97.8), 93.9% (95% CI, 87.6–97.1) and 91.3% (81.5–96), respectively. The causes of amputation in nine patients were infection (*n* = 4) and local recurrence (*n* = 5). The mean function score in 96 patients according to the Musculoskeletal Tumor Society classification system [7] was 23.7% (79 %).

## 4. Discussion

We elucidated the long-term follow-up outcomes of the KMLS systems in the distal femur. When we included all causes of implant failure, implant survival rates at 5, 10, and 15 years in 125 patients were 72%, 58.5%, and 39.4%, respectively. Metal removal (major revision), including the femoral component, stem and tibial components, was performed in 56 patients. The implant survival rates at 5, 10, and 15 years (major revision) were 72.8%, 64.9%, and 46.3%, respectively. Although no significant differences in implant survival were observed between cementless and cement stem fixation, aseptic loosening was likely observed in patients with cement fixation and stem breakage in patients with cementless fixation. Amputation was required in nine patients (7.2%).

In this study, we estimated the implant survival rate according to the content of revision surgery (any or metal removal) because other long-term follow-up studies excluded minor procedures, such as bushing exchanges for the knee hinge axle, which were not considered implant failure [8,9,10,11,12] (Table 4). Our results are generally consistent with the findings of reports published in the literature [8,9,10,11,12]. In this study, we aimed to compare implant survival and the cause of failure between cement and cementless stem fixation.

In this study, aseptic loosening occurred in 12% of the cases and ranged from 4.9% to 21% in other long-term follow-up studies [8,9,10,11,12]. Specifically, aseptic loosening was observed in six patients (8.2%) with cementless fixation and nine patients (17.3%) with cement fixation. We were unable to demonstrate an influence of the ratio of total length of prosthesis (TLP)/stem length (SL) and femoral resection length in addition to other clinical variables, although Batta et al. showed that a large TLP/SL and femoral resection length significantly increased the rate of loosening [10]. However, loosening appeared to be a specific problem in the implants that were used as a revision that previously failed due to loosening. In this study, loosening recurred in 7 of 15 patients, while aseptic loosening recurred in seven patients. Cementless fixation is expected to reduce the risk of aseptic loosening because cementless fixation can achieve bone ingrowth, resulting in long-term stability [13,14]. In addition, a rotating hinge, which is applied to the PHK III and KMLS systems, allows a wider range of movement and a lower torsional force, which should reduce the risk of aseptic loosening [5,9,11,15]. In this study, although no significant differences were observed in the rate of loosening between cement and cementless stems, it was likely that aseptic loosening would be observed in patients with cement fixation at 15 years after surgery.

We have to refer to the rate of stem breakage (11.2%), which might be higher than those reported in other long-term follow-up studies (2–5.8%) [8,9,10,11,12]. In this study, stem breakage was observed in patients treated with cementless fixation. Furthermore, the cumulative incidence rate continuously increased in patients with cementless fixation. We consider that the femoral cementless stem design may have affected the risk of implant breakage. Stem breakage occurred mainly through the most distal non-penetrating holes in the cementless stem in the KMLS system, suggesting the convergence of mechanical stress in the most distal partial holes [6]. Stem design in the Kotz Modular Femoral Tibial Replacement (KMFTR) has also been reported to predispose patients to breakage [14,16]. We have already developed a press-fit stem with a taper-shaped design. Additionally, stem breakage was observed in patients with smaller femoral stem diameters. Specifically, 10 and 11 mm of stem fixed with cement and 12 mm of the cementless stem should be carefully followed up for stem breakage for a long time.

Infection occurred in 10.4% of patients, comparable to other long-term follow-up studies [8,9,10,11,12]. In this study, patients who resected three or all femoral quadriceps muscles with tumors were likely to develop an infection. Providing adequate soft tissue coverage after reconstruction may be a critical factor in reducing infection [11,17]. Therefore, a musculocutaneous flap after resection of three or all femoral quadriceps muscles with tumors may be a useful option to reduce infection. Unlike previous studies, we did not find a relationship between sex, BMI, and infection in this study.

Local recurrence is a threatening complication that can result in revision or amputation [18,19]. In this study, the limb salvage rate was consistent with previous reports, and the cause of amputation was due to infection and local recurrence.

There were some limitations to this study. It is difficult to adequately compare implant survival rates between KMLS and other systems because different classifications and definitions of implant failure have been used. The retrospective nature of this study was another limitation.

## 5. Conclusions

The KMLS systems represent a reliable system with long-term results. Stem breakage should be considered in patients with cementless and/or smaller femoral stem sizes. Aseptic loosening should be considered in patients with cement systems after 10 years.

## Figures and Tables

**Table 1:** Patient backgrounds.

Characteristics	Parameter	*N*
Age (years)	Mean	35
	Range	9–79
Sex (*N*)	Male	59
	Female	66
Perioperative cx (*N*)	Yes	84
	No	41
Femoral stem fixation (*N*)	cement-type	52
	cementless-type	73
Femoral component (*N*)	90 mm	7
	110 mm	19
	130 mm	23
	150 mm	30
	170 mm	19
	190 mm	8
	>210 mm	19
Stem length (*N*)	130 mm	120
	170 mm	5
TLP/SL (ratio)		1.65–3.23
Stem diameter (*N*)	cement-type	
	10 mm	4
	11 mm	26
	12 mm	12
	13 mm	10
	cementless-type	
	10 mm	1
	11 mm	1
	12 mm	46
	13 mm	14
	14 mm	7
	15 mm	4

cx: chemotherapy, TLP/SL: ratio of total length of prostheses/stem length.

**Table 2:** Cause of implant failure.

Complications	*N*
Aseptic loosening	15
Stem breakage	14
Infection	13
Rotator-hinge bushing failure	13
Local recurrence	5
Fracture	2
Others	3

**Table 3:** The relationship between implant survival and patient characteristics.

Variables		*N*	Implant Survival Rate (%)			
			5-Years	10-Years	15-Years	*p* Value
Age	<20 years	52	71.2	60.9	37.3	0.96
			(56.8–81.5)	(46.1–72.8)	(22.1–52.6)	
	>20 years	73	72.5	56.2	42.9	
			(60.7–81.3)	(43–67.5)	(27.8–57.2)	
BMI	>25 kg/m^2^	13	61.5	41	41	0.93
			(30.8–81.8)	(8.5–72.5)	(8.5–72.5)	
	<25 kg/m^2^	83	71.4	58.1	35.6	
			(60.4–79.8)	(46.2–68.3)	(21.8–49.7)	
Sex	Male	59	71.1	57.5	41.2	0.73
			(57.7–80.9)	(43.5–69.3)	(25.7–56)	
	Female	66	72.7	59.3	37.5	
			(60.3–81.9)	(45.7–70.6)	(22.1–52.8)	
Perioperative	Yes	83	68.6	56	33.1	0.18
			(57.4–77.4)	(44.2–66.2)	(19.9–46.9)	
chemotherapy	No	42	78.6	63.9	52.1	
			(62.9–88.2)	(46.4–77)	(33.7–67.7)	
Patella	Yes	21	66.7	55.4	47.5	0.92
			(42.5–82.5)	(31.4–74)	(23.3–68.3)	
replacement	No	104	73.1	59.3	37.2	
			(63.4–80.6)	(48.7–68.4)	(24.8–49.7)	
Resection of	Extra-capsule	21	66.3	63.4	41.6	0.11
			(42–82.3)	(52.4–72.5)	(28.3–54.4)	
Joint	Intra-capsule	94	72.3	38.3	38.3	
			(62.1–80.2)	(17.1–59.3)	(17.1–59.3)	
Resection	3 to 4	12	66.7	57.1	28.6	0.83
			(33.7–86)	(25.4–79.6)	(4.9–59.4)	
of quadriceps femoris	0 to 2	96	71.8	58.9	41.4	
			(61.7–79.7)	(47.8–68.4)	(28.4–53.8)	
Fixation of	Cementless	73	76.7	55.7	35.3	0.82
			(65.2–84.8)	(42.9–66.8)	(21.7–49.3)	
femoral stem	Cement	52	65.2	63.1	46.4	
			(50.6–76.5)	(48.3–74.6)	(28.8–62.4)	

**Table 4:** The long-term (Mean follow-up duration > 10 years) clinical outcome in patients with prosthetic replacement at distal part of the femur.

Ref	*N*	Stem	Hinge	Implant survival			Complications		
		Fixation	Type	5-years	10-years	15-years	Loosening	Infection	F-stem b
[8]	152	C	modular; RH	74% *	59% *	50% *	21%	9.9%	2%
			custom; FH						
[9]	669 ^#^	C 9%	FH		80% *		5.7%	8.2%	4.4%
		C-less 91%							
[10]	69	C-less	RH	73% *	65% *	55% *	13%	7.2%	4.3%
[11]	335	C 53%	FH; 48%	83% *	67% *	51% *		9.6%	2.1%
		C-less 47%	RH; 52%						
[12]	93	C	RH		73.3% *	62.8% *	4.9%	11.7%	5.8%
Ours	125	C 42%	RH	72.8% *	64.9% *	46.3% *	12%	10.4%	11.2%
		C-less 58%		(72% **)	(58.5% **)	(39.4% **)			

FH: fixed hinge, RH: Rotating hinge, F-stem b: Femoral stem breakage, C: Cement, C-less: Cementless, *: Minor procedures such as bushing exchanges for the knee hinge axle were not considered implant failure, **: Minor procedures were considered implant failure. #71% of 669 patients underwent distal femur replacement.

## Data Availability

No new data were created or analyzed in this study. Data sharing is not applicable to this article.
